# Shelf humic substances as carriers for basin-scale iron transport in the North Pacific

**DOI:** 10.1038/s41598-020-61375-7

**Published:** 2020-03-11

**Authors:** Youhei Yamashita, Jun Nishioka, Hajime Obata, Hiroshi Ogawa

**Affiliations:** 1 Faculty of Environmental and Earth Science, Hokkaido University, Sapporo, Japan; 2 Pan-Okhotsk Research Center, Institute of Low Temperature Science, Hokkaido University, Sapporo, Japan; 3 Atmosphere and Ocean Research Institute, The University of Tokyo, Kashiwa, Japan

**Keywords:** Biogeochemistry, Marine chemistry

## Abstract

Iron is one of the key elements controlling phytoplankton growth in large areas of the global ocean. Aeolian dust has traditionally been considered the major external source of iron in the North Pacific. Recent studies have indicated that sedimentary iron from the shelf region of the Sea of Okhotsk has a strong impact on the iron distribution in the North Pacific, while the mechanism supporting its long-distance transport remains poorly understood. Here, we report that refractory shelf humic substances, which complex and carry dissolved iron, are transported conservatively at least 4000 km from the shallow sediments of the Sea of Okhotsk to the subtropical North Pacific with the circulation of intermediate water. This result indicates that shelf humic substances are probably one of the key factors shaping the distribution of dissolved iron in the ocean interior.

## Introduction

Iron (Fe) is one of the essential elements for marine life and has low solubility in oxic seawater^1,2^; therefore, external inputs of Fe influence ocean primary productivity^3,4^. Aeolian dust, shelf sediments, and hydrothermal vents are major external sources of dissolved Fe (Fe_d_), and the mechanisms that make Fe soluble and contribute to long-distance transport are vital to connecting external sources with primary productivity in remote ocean areas^3,5^. Although aeolian dust has traditionally been considered the major external source of Fe to the ocean^6,7^, shelf sediments have been noted to be much more important than aeolian dust or hydrothermal vents in terms of the percentage of the Fe_d_ inventory in the ocean and its role in fueling the biological carbon pump^5^. The chemical species of Fe_d_ contributing to long-distance transport from sediments are thus critical information to understand not only marine Fe cycle but also global carbon cycle.

Organic ligands increase the capacity of Fe to dissolve in seawater by complexing with Fe and possibly contribute to long-distance transport through protecting Fe_d_ from being scavenged^1–4^. Siderophores, saccharides, and humic substances have been considered probable Fe-binding organic ligands in marine environments^4,8,9^. Among these substances, refractory humic substances are probably the most important Fe_d_ carriers in the subsurface ocean because siderophores and saccharides are microbiologically labile^10,11^. Humic substances, which are complex and heterogeneous mixtures of organic molecules that form during the decay and transformation of biogenic remains, are highly functionalized and are generally characterized by their color due to their ultraviolet-visible (UV-Vis) absorbance^12^. As a consequence of their absorbance characteristics, humic substances exhibit fluorescence properties commonly referred to as humic-like fluorescent dissolved organic matter (FDOM_H_)^13,14^. It is well established that FDOM_H_ is universally distributed over the Earth’s surface, namely, from streams to deep oceans^15,16^. Fe-binding ligands^4,17^ and FDOM_H_^18–20^ have been reported to be released during the microbial degradation of sinking particles, and a linear relationship was found between FDOM_H_ and Fe(III) solubility (dissolution capacity of Fe) in subsurface waters^21–24^; thus, FDOM_H_ is very likely a major Fe-binding organic ligand in the dark ocean. A global ocean Fe biogeochemical model also successfully applied autochthonous FDOM_H_ as the main organic ligand to reproduce the Fe_d_ distribution in the ocean^25^. However, oceanographic linkage between Fe_d_ and FDOM_H_ has not been explored with the basin-scale.

**Figure 1:**
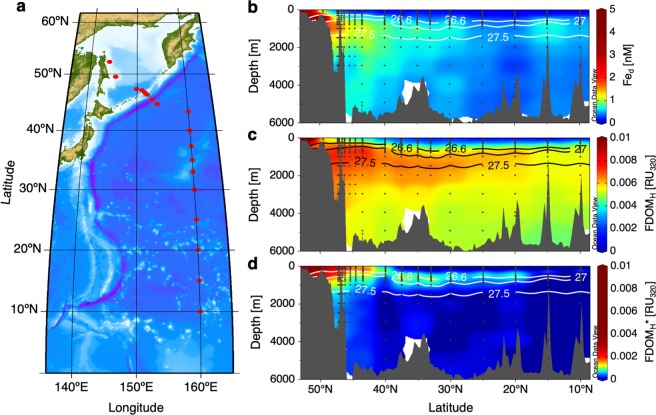
Basin-scale distributions from the shelf of the Sea of Okhotsk to the subtropical North Pacific. (**a**) Station locations. (**b**) Fe_d_ (nM). (**c**) FDOM_H_ (RU_320_). (**d**) FDOM_H_* (RU_320_). Solid lines in (**b**–**d**) represent the 26.6σ_θ_, 27.0σ_θ_, and 27.5σ_θ_ contours, and 26.6–27.0σ_θ_ and 27.0–27.5σ_θ_ correspond to upper and lower intermediate water, respectively.

Here, we present the distribution of Fe_d_ together with FDOM_H_ along a section in the western North Pacific (Fig. 1a) where basin-scale transport of sedimentary Fe_d_ from the shelf region of the Sea of Okhotsk has been reported^26,27^. We hypothesize that sedimentary Fe_d_ complexed with FDOM_H_ is stable and contributes to long-distance transport with the circulation of intermediate water. Therefore, we separate the allochthonous (shelf-derived) fraction of FDOM_H_ from the autochthonous fraction, which was determined by the relationship with apparent oxygen utilization (AOU)^18–20^, and identify the relative contribution of allochthonous FDOM_H_ as a carrier of Fe_d_ in the intermediate water of the western North Pacific.

## Results

### Transport of allochthonous FDOM_H_ by intermediate water

Analogous to previous studies^26,27^, this study found the highest concentrations of Fe_d_ evident in the shelf region of the Sea of Okhotsk, and high concentrations of Fe_d_ were measured in intermediate to deep waters around the Bussol’ Strait due to strong diapycnal tidal mixing (Fig. 1b). The diapycnal tidal mixing at the deep sill of the Bussol’ Strait (2200 m) is known to be important to determine the physical and chemical properties of the intermediate water^28–30^. The levels of Fe_d_ in the North Pacific Intermediate Water (NPIW; 26.6–27.5σθ)^31^, which is characterized by a salinity minimum in subtropical regions (Supplementary Fig. 1a), were higher than those in the upper/deeper water masses. It has been suggested that the Fe_d_ derived from shelf sediments in the Sea of Okhotsk is transported to the basin region by the Okhotsk Sea Intermediate Water (OSIW; 26.6–27.0σ_θ_)^32^ and then spreads through the circulation of intermediate water, including the NPIW, in the North Pacific^26,27^.

The lowest level of FDOM_H_ was observed in surface waters, which was likely due to the photobleaching of FDOM_H_^33–35^, except in the shelf region of the Sea of Okhotsk (Fig. 1c). The levels along the transect generally increased with depth in the mesopelagic layer (200–1000 m) and then slightly decreased with depth in the deep layer (>1000 m). The distribution pattern of FDOM_H_ was almost identical to that of AOU (Supplementary Fig. 1b), as previously reported^18–20,36,37^.

**Figure 2:**
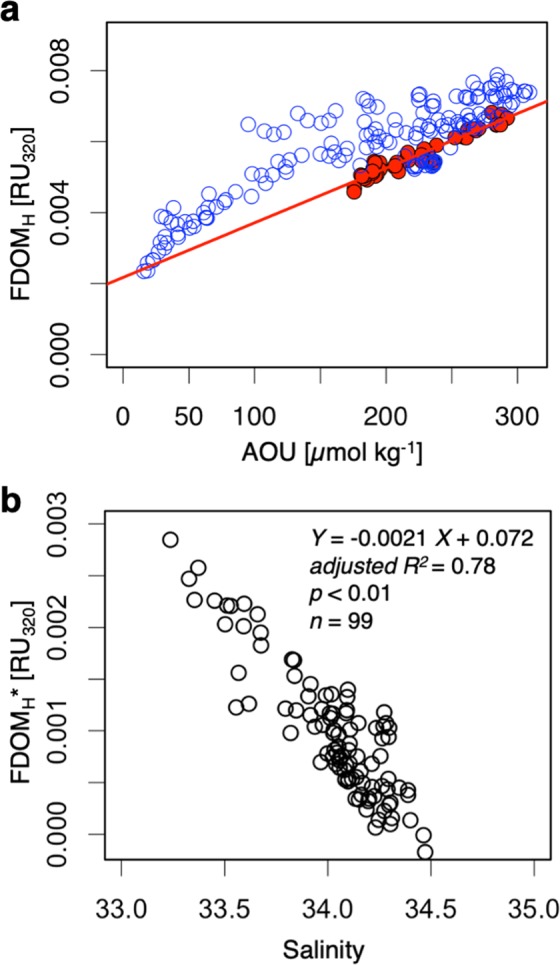
Allochthonous FDOM_H_ (FDOM_H_*) in the intermediate water. (**a**) Relationship between AOU and FDOM_H_ in the deep layer (>1000 m) of the 160 °E transect (red circles) and in the mesopelagic layer (200–1000 m) from the basin of the Sea of Okhotsk to the subtropical North Pacific (blue circles). The red line represents the linear regression of the deep layer. (**b**) Relationship between salinity and FDOM_H_* in the intermediate water. The samples in the water masses with a density range of 26.6–27.5σ_θ_ and depths of greater than 200 m from the basin of the Sea of Okhotsk to the subtropical North Pacific (20 °N) are plotted.

Interestingly, however, the FDOM_H_-AOU relationships in the mesopelagic layer and the deep layer were different (Fig. 2a). The FDOM_H_ levels in the mesopelagic layer were higher than those in the deep layer, thus showing deviations from the linear regression line obtained for the deep layer. Similar but smaller deviations in mesopelagic FDOM_H_ from the deep linear regression line have also been observed in the central North Pacific^18^. Because AOU represents the amount of oxygen consumed by respiration after the subduction of a water mass, the deep linear regression line has been attributed to the *in situ* FDOM_H_ produced by microbes during the oxidation of organic matter^18–20^. Thus, the autochthonous fraction of FDOM_H_ in the mesopelagic layer corresponds to the linear portion of the regression between AOU and FDOM_H_ (determined for the deep layer); then, the contribution of allochthonous FDOM_H_, which is defined here as FDOM_H_*, can be estimated quantitatively (see Methods).

The distribution pattern of FDOM_H_* was distinctly different from that of FDOM_H_ (Fig. 1c,d). The highest level was observed in the shelf region of the Sea of Okhotsk. The levels of FDOM_H_* in the OSIW and the upper intermediate water (26.6–27.0σ_θ_)^31^ were higher than those in the upper/deeper water masses from the Sea of Okhotsk to the south as far as 20 °N in the subtropical North Pacific gyre, corresponding to the southernmost region of the NPIW distribution^31^. FDOM_H_* accounted for 37 ± 7% (*n* = 4) and 12 ± 4% (*n* = 9) of the bulk FDOM_H_ in the OSIW and the upper NPIW at 20–30 °N, respectively (Supplementary Fig. 2). A negative linear relationship was evident between FDOM_H_* and salinity in the intermediate water (adjusted *R*^2^ = 0.78, Fig. 2b), even though FDOM_H_ was not linearly related to salinity in the intermediate water (adjusted *R*^2^ = 0.003). Because the OSIW, which is influenced by the dense shelf water that forms in the coastal polynya through sea-ice formation involving the interaction with sediments^32^, greatly contributes to the formation of the upper intermediate water^31^, its negative relationship with salinity indicates that FDOM_H_* from the shelf sediments of the Sea of Okhotsk is conservatively transported across the North Pacific through the formation and circulation of the intermediate water. The residence time of the OSIW in the Sea of Okhotsk was estimated to be 1.4–7 years^38,39^. The apparent ages of intermediate water, including the NPIW, from the western subarctic to the subtropical North Pacific gyre were suggested to be ~25 years^40^. Such timescales of the circulation of the intermediate water indicate that FDOM_H_* is not removed nor transformed in the dark ocean for at least several decades.

### Role of FDOM_H_ in the chemical speciation of Fe_d_

The Fe(III) solubility has been found to be controlled by organic complexation^1,2^; thus, Fe(III) solubility is not simply related to FDOM_H_ level in surface waters where siderophores and saccharides in addition to FDOM_H_ are possibly key organic ligands of Fe_d_^21,23,41,42^. However, it has been reported that the FDOM_H_ level is linearly related to the Fe(III) solubility throughout the water column, except in the surface water^21–23^. The linear relationship did not differ between the deep layer and the mesopelagic layer in the Sea of Okhotsk and the western subarctic Pacific^21^, where autochthonous FDOM_H_ is dominant and where FDOM_H_* co-occurs with autochthonous FDOM_H_. Such a relationship indicates that Fe(III) solubility represented by the FDOM_H_ level is the same between allochthonous and autochthonous fractions in the region. Therefore, we can estimate the Fe(III) solubility of bulk FDOM_H_, as well as FDOM_H_*, based on a linear relationship between Fe(III) solubility and FDOM_H_ level (see Methods).

**Figure 3:**
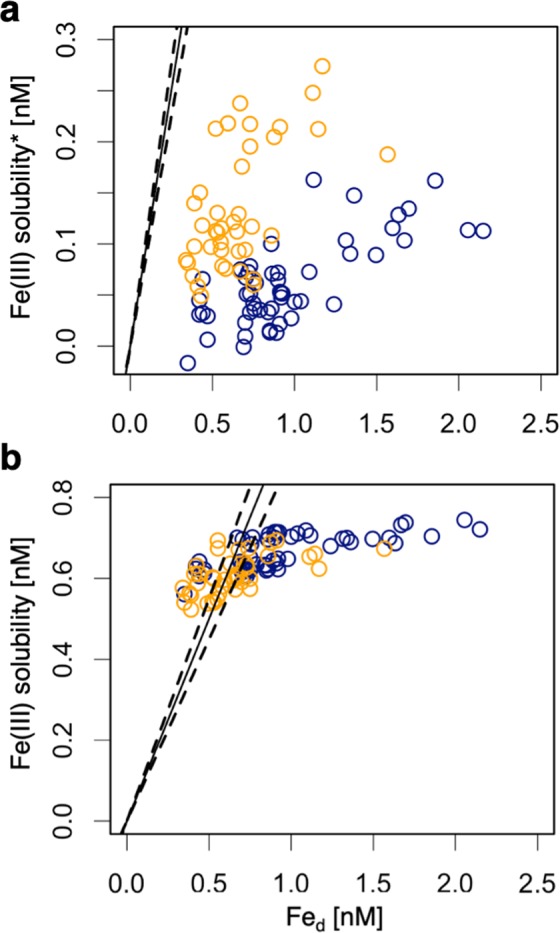
Relationships between Fe_d_ concentration and Fe(III) solubility. (**a**) Fe_d_ concentration versus Fe(III) solubility* estimated from FDOM_H_*. (**b**) Fe_d_ concentration versus Fe(III) solubility estimated from FDOM_H_. The samples in the upper intermediate water (26.6–27.0σ_θ_, orange circles) and lower intermediate water (27.0–27.5σ_θ_, blue circles) from the basin of the Sea of Okhotsk to the subtropical North Pacific (20 °N) are plotted. Solid lines and dotted lines indicate the 1:1 line of Fe_d_ concentration versus its solubility and standard deviation of the 1:1 line, respectively.

The Fe(III) solubility of FDOM_H_* (Fe(III) solubility*) was lower than the corresponding Fe_d_ concentration in the upper intermediate water and the lower intermediate water (27.0–27.5σθ) (Fig. 3a). The majority of the flux of Fe_d_ from sediments to the water column has been considered to be dominated by organic-Fe(III) complexes^43,44^. Thus, the relationship (Fig. 3a) indicates that a specific fraction of Fe_d_ from shelf sediments occurs as Fe_d_ complexed with FDOM_H_* and is transported across the North Pacific with the conservative spread of FDOM_H_*. This mechanism is effective for the long-distance transport of Fe_d_, particularly in the mesopelagic and deep layers where FDOM_H_ is not degraded by sunlight.

The other fractions of Fe_d_, namely, excess Fe_d_ compared with corresponding Fe(III) solubility* (Fig. 3a), are not complexed with FDOM_H_*. The Fe_d_ concentration also exceeded the Fe(III) solubility of bulk FDOM_H_, particularly in the OSIW, as well as in the lower intermediate water in the Sea of Okhotsk and the western subarctic Pacific gyre (~40 °N) (Figs. 1b and 3b), suggesting that some fractions of Fe_d_ are complexed with neither FDOM_H_* nor autochthonous FDOM_H_. Similar to our observations, excess Fe_d_ concentrations compared with its bulk solubility have been reported in the mesopelagic and deep layers of the western subarctic Pacific gyre^23^. The Fe(III) solubility was obtained by measuring Fe in the soluble fraction (<0.025 µm)^21–23^. Because the molecular weight of FDOM_H_ is reported to be less than 1.8 kDa^34^, quite smaller than 0.025 µm, soluble Fe can form complexes with humic substances, as indicated by FDOM_H_. These pieces of evidence indicate that excess Fe_d_ compared with the solubility derived from bulk FDOM_H_ can occur as colloidal Fe (0.025–0.22 µm), which is not complexed with FDOM_H_. Although the size fractionation was not determined in this study, the substantial occurrence of colloidal Fe has been observed in the intermediate water of the western subarctic Pacific^45^, which is the same water mass observed in this study.

## Discussion

**Figure 4:**
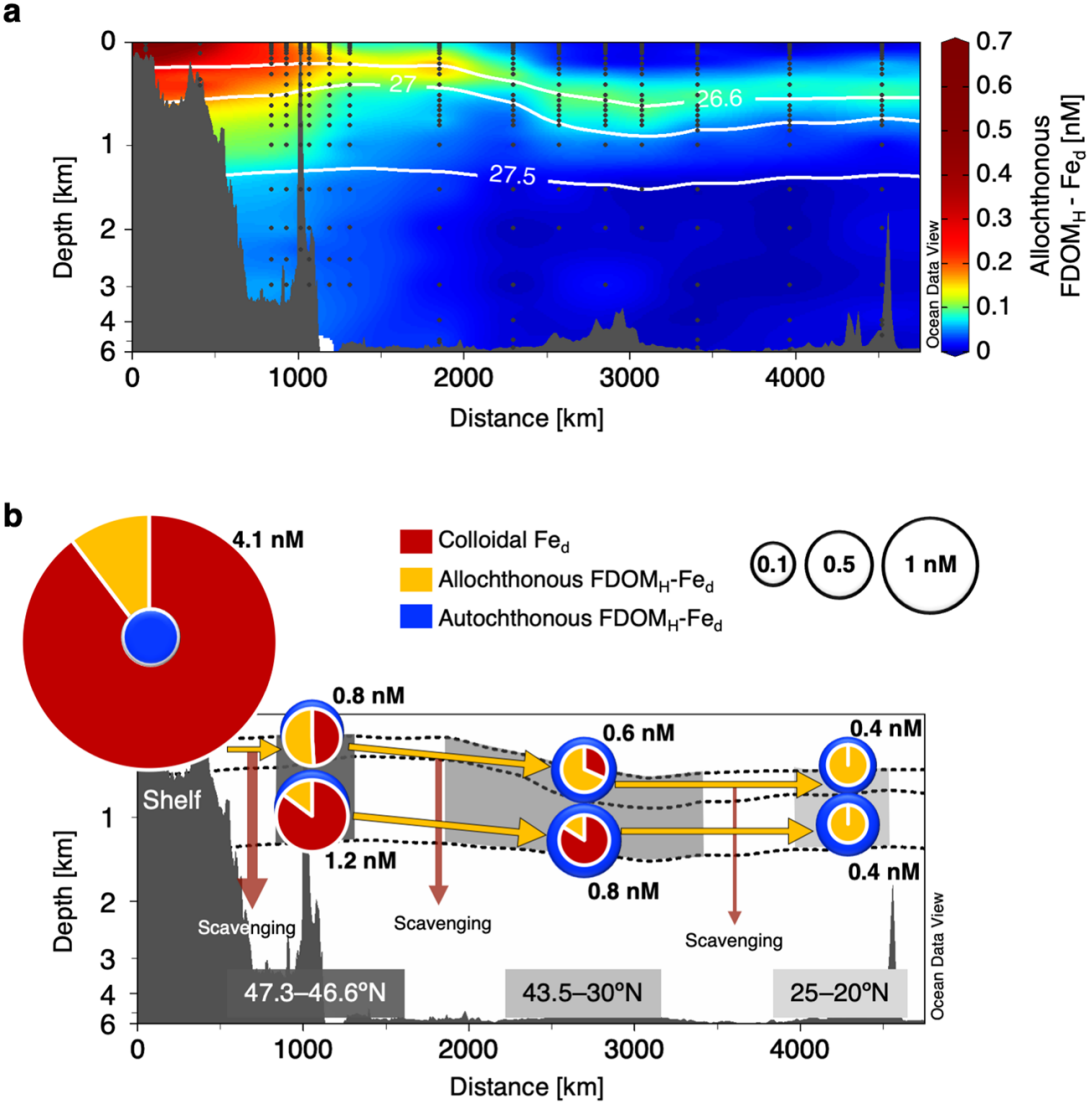
Basin-scale transport of sedimentary Fe_d_ by the complexation with allochthonous FDOM_H_. (**a**) Distribution of allochthonous FDOM_H_-Fe complexes from the northernmost station on the shelf of the Sea of Okhotsk to the subtropical North Pacific (20 °N). (**b**) Illustration of the chemical forms of Fe_d_ during transport by the circulation of upper and lower intermediate waters. The values of 0–2 km are stretched out on the y-axes. The solid white lines in (**a)** and dotted black lines in (**b**) represent the 26.6σ_θ_, 27.0σ_θ_, and 27.5σ_θ_ contours, and 26.6–27.0σ_θ_ and 27.0–27.5σ_θ_ correspond to upper and lower intermediate water, respectively. Pie diagrams in (**b**) show the average relative contributions of allochthonous FDOM_H_-Fe complexes and colloidal Fe. Concentric circles represent average concentrations of allochthonous FDOM_H_-Fe complexes + colloidal Fe (warm colors) and autochthonous FDOM_H_-Fe complexes (cold colors). Note that the two circles for upper and lower intermediate waters located at 47.3–46.6 °N are illustrated non-concentrically because the concentrations of the two fractions are almost the same. The concentrations described near the circles represent the average Fe_d_ concentrations.

FDOM_H_*, namely, allochthonous FDOM_H_, is most likely supplied from sediments as stable complexes with Fe_d_ since major forms of sediment-derived Fe_d_ are organic complexes^43,44^. Assuming that the other Fe_d_ preferentially forms complexes with autochthonous FDOM_H_ in the intermediate and deep waters, the spatial distribution of Fe_d_ concentrations (Fig. 1b) can be separated into three groups (Fig. 4 and Supplementary Fig. 3). High concentrations of allochthonous FDOM_H_-Fe complexes and colloidal Fe occur in the shelf region of the Sea of Okhotsk and spread to the western North Pacific through circulation of intermediate water, including the NPIW. The allochthonous FDOM_H_-Fe complexes and colloidal Fe are mainly distributed in the upper and lower intermediate waters, respectively, implying that the allochthonous FDOM_H_-Fe complexes can make more important contributions to primary production in remote areas due to intrusion into the upper layer. Interestingly, a shift in dominant groups of sedimentary Fe_d_ from colloidal Fe to allochthonous FDOM_H_-Fe complexes involving a dramatic decrease in Fe_d_ concentration was evident during transport by the OSIW in the Sea of Okhotsk. The relative contribution of allochthonous FDOM_H_-Fe complexes was 10 ± 5% in the OSIW on the shelf of the Sea of Okhotsk and changed to 51 ± 15% in the upper intermediate water around the Bussol’ Strait. This result is consistent with the results of a previous study that estimated that 76% of sedimentary Fe_d_ is scavenged during transport from the shelf to the basin region in the Sea of Okhotsk^46^. The major groups of Fe_d_ continuously shifted and reached 100% allochthonous FDOM_H_-Fe complexes at 25–20 °N along with circulation of upper intermediate water, including the NPIW, indicating that the conservative behavior of allochthonous FDOM_H_ contributes to long-distance transport of sedimentary Fe_d_ over more than 4000 km to the subtropical North Pacific.

Relatively high concentrations of colloidal Fe and autochthonous FDOM_H_-Fe complexes were observed in and below the lower intermediate water in the subarctic gyre (Supplementary Fig. 3). Such high concentrations may be explained by scavenged sedimentary Fe_d_ occurring as a result of reversible Fe exchange processes, including stabilization by organic ligands in the dissolved phase, aggregation and disaggregation of nanoparticles, and sinking of aggregated nanoparticles^47,48^. The colloidal Fe was greater than allochthonous FDOM_H_-Fe in the lower intermediate water from the source region to 30 °N (~3500 km of transport distance) but was completely replaced with allochthonous FDOM_H_-Fe complexes in the lower NPIW at 25-20 °N. These results suggest that reversible Fe exchange processes are effective for long-distance transport of sedimentary Fe_d_, similar to hydrothermal vent systems^47^, but they are not as effective as complexation with allochthonous FDOM_H_. Although dissolved organic matter (DOM) complexation with soluble Fe_d_ has not yet been understood for hydrothermal plumes^47^, this study clarifies that the conservative behavior of allochthonous FDOM_H_ can contribute to the long-distance transport of sedimentary Fe_d_ in the subsurface ocean.

The transport mechanism of sedimentary Fe_d_ reported in this study can be applied to the western Arctic Ocean, where high levels of Fe_d_ and FDOM_H_ are evident in dense shelf water^49,50^. It has been documented that hypoxic shallow sediments are an important source of Fe_d_ and labile particulate Fe through the supply of Fe(II) from the sediments, oxidation to Fe(III), and chelation of Fe(III) with organic ligands or formation of inorganic Fe(III) to labile particles^43^. It has also been reported that FDOM_H_ is produced in marine sediments even under anoxic conditions^51^. Therefore, it can be concluded that FDOM_H_ are primary organic ligands contributing to the long-distance transport of sedimentary Fe for the whole ocean, although stable transport is limited to the dark ocean where photodegradation of FDOM_H_ is inhibited. An application of the method used in this study to other intermediate water systems will clarify the generality regarding with the relationship between Fe(III) solubility and FDOM_H_ as well as the role of FDOM_H_ as a carrier of sedimentary Fe.

Apart from macronutrients, the chemical framework of the Fe cycle in the ocean has not been well established because Fe has extremely low solubility in modern seawater. The chemical properties of Fe control input from external sources and its residence time, which shape the Fe distribution in the ocean. Although organic ligands have been considered a major factor increasing Fe solubility, the exact role of organic complexation in the Fe cycle, and in fact the very nature of the ligands that stabilize soluble Fe, have been incompletely characterized. This study clearly indicates that FDOM_H_ is a factor that controls the residence time of Fe_d_, at least sedimentary Fe_d_. Although aeolian dust has traditionally been considered a major source of Fe for phytoplankton growth in the western North Pacific, the episodic inputs of aeolian dust may not be sufficient to sustain primary productivity in the region^52^. The stable transport of sedimentary Fe_d_ complexed with allochthonous FDOM_H_ by intermediate water possibly influence primary productivity in a wide area of the western North Pacific. Thus, FDOM_H_ can be a crucial factor controlling the Fe cycle in the ocean.

Allochthonous and autochthonous FDOM_H_, as ligands of Fe_d_, are able to be determined by salinity and AOU in the western North Pacific (Fig. 2). A global ocean Fe biogeochemical model successfully parameterized autochthonous FDOM_H_ as the main ligand^25^. A parameterization of allochthonous and autochthonous FDOM_H_ in the biogeochemical models may result in the accurate reproduction of the modern ocean Fe cycle and consequently ocean ecosystems and carbon cycling, which will have implications for the appropriate estimation of how climate change will affect ocean productivity^3,4^.

## Methods

### Oceanographic observations

Observations in the western North Pacific were conducted along the 160 °E transect in July 2012 as part of the R/V *Hakuho Maru* cruise (KH-12-3). Observations from the basin of the Sea of Okhotsk to the western subarctic Pacific gyre through the Bussol’ Strait and the shelf region of the Sea of Okhotsk were conducted in June 2014 by the R/V *Professor Multanovskiy* and in August 2006 by the R/V *Professor Khromov*, respectively. Salinity and temperature were measured using a conductivity-temperature-depth (CTD) sensor, and dissolved oxygen (DO) concentrations were measured using an oxygen sensor connected to a CTD. The DO concentrations were also measured on board by the Winkler titration method, and the DO concentrations measured by the sensor were calibrated using the concentrations determined by the Winkler method. The oxygen solubility was calculated using the function of Weiss (1970)^53^, and apparent oxygen utilization (AOU) was then calculated as the difference between the solubility and the measured DO concentration. Seawater from the surface to bottom layers (16–29 depths) was collected with acid-cleaned Teflon-coated 10- or 12-L Niskin-X bottles that were mounted on the CTD with a carousel multi-sampling system during the R/V *Hakuho Maru* and R/V *Professor Multanovskiy* cruises. The sampling method used for seawater from the two stations (C3 and B5) located in the shelf region of the Sea of Okhotsk during the R/V *Professor Khromov* cruise has been described elsewhere^46^.

### Dissolved iron

Concentrations of dissolved iron (Fe_d_) in the shelf region of the Sea of Okhotsk measured during the R/V *Professor Khromov* cruise were derived from previously reported data^46^. To collect a subsample from the Niskin-X sampler during the R/V *Hakuho Maru* (KH-12-3) cruise, the sampler was transported in a clean air bubble (filled with air that had been passed through a high-efficiency particulate air filter) and a 0.2-μm Acropak filter (Pall Corporation) was connected to the Niskin-X spigot; the filtrate was then collected in acid-cleaned 125-mL low density polyethylene (LDPE) bottles (Nalgene Co., Ltd). To collect a subsample from the Niskin-X sampler during the R/V *Professor Multanovskiy* cruise, the sampler was placed in a clean tent and a 0.22-μm Millipak filter (Millipore Corporation) was connected to the Niskin-X spigot; the filtrate was then collected in acid-cleaned 125-mL LDPE bottles (Nalgene Co., Ltd). We confirmed that there were no significant differences between the Fe_d_ concentrations measured using the Acropak filter and the Millipak filter.

The filtrate (<0.22 μm) was adjusted to pH <2 by the addition of ultrapure HCl (Tamapure AA-10, final HCl concentration of the sample was 0.024 M) and then allowed to remain for one to three months at room temperature in the onboard clean room. Each sample was then adjusted to pH 3.2 just before its measurements by the addition of ammonium solution and a formic acid (10 M)–ammonium (2.4 M) buffer. Fe_d_, defined as the leachable Fe in the filtrate at pH <2, was then analyzed in the onshore laboratory using a flow injection analysis (FIA) chemiluminescence detection system^54^. All sample treatments were performed under laminar flow in the onboard or onshore clean air laboratory.

The Fe_d_ measurements and reference seawater analyses in this study were quality controlled using SAFe (Sampling and Analysis of Iron) cruise^55^ reference standard seawater (obtained from the University of California Santa Cruz for an inter-comparison study). We measured a SAFe reference sample during every sample measurement run of the FIA instrument performed in the onboard and onshore laboratories. The consensus values for Fe(III) in the SAFe reference standard seawater are 0.093 ± 0.008 nM (S) and 0.933 ± 0.023 nM (D2) (May 2013, www.geotraces.org), and we obtained values of 0.098 ± 0.010 nM (n = 12) (S) and 0.976 ± 0.101 nM (n = 10) (D2) using our method. This good agreement demonstrates that our data quality was high and that our data are comparable with the global GEOTRACES dataset. The detection limit (three times the standard deviation of the Fe(III) concentration of purified seawater (0.036 nM) that had been passed through an 8-quinolinol resin column three times to remove Fe) was 0.020 nM.

### Humic-like fluorescent dissolved organic matter

To determine the level of humic-like fluorescent dissolved organic matter (FDOM_H_) in the samples obtained during the R/V *Hakuho Maru* and R/V *Professor Multanovskiy* cruises, the seawater samples from the Niskin-X sampler were poured directly into pre-combusted, triple-rinsed glass vials with Teflon-lined caps. The glass vials were thoroughly washed with Milli-Q water for their next use on board the ship. Just after sampling, the seawater was allowed to stand until reaching room temperature without undergoing any filtration procedure, and fluorescence measurements were performed with a spectrofluorometer (RF-1500, Shimadzu) with a 1-cm quartz cell. The fluorescence intensity of the FDOM_H_ was determined at excitation and emission wavelengths of 320 nm and 420 nm, respectively, according to Yamashita *et al*.^37^. It was reported that the observed differences in FDOM_H_ levels with and without filtration using GF/F glass fiber filters were negligible for the open ocean samples^37^.

Seawater samples collected at two stations located in the shelf region of the Sea of Okhotsk during the R/V *Professor Khromov* cruise were filtered with a 0.22-μm Millipak filter (Millipore Corporation) connected to the Niskin-X spigot and poured into acid-cleaned fluorinated high-density polyethylene (HDPE) bottles (Nalgene Co., Ltd). The filtrate was stored frozen in the dark until analysis. The frozen samples were thawed and allowed to stand until reaching room temperature; fluorescence measurements were then conducted as described above.

After the analysis, the fluorescence intensities were corrected to the area under the water Raman peak of Milli-Q water (excitation = 320 nm), which was analyzed daily with freshly prepared Milli-Q water and calibrated to Raman Units (RU_320_)^56^. Because the instrument-specific response^57^ of the spectrofluorometer (RF-1500, Shimadzu) was not corrected commercially, the instrument-specific response was corrected with the comparison of FDOM_H_ fluorescence intensity in RU_320_ obtained by an instrument-specific response-corrected spectrofluorometer (FluoroMax-4, Horiba)^58^. The conversion factor from RU_320_ to commonly used Raman Units (RU; fluorescence intensity corrected by peak area of Raman scatter at 350 nm)^56,58^ was 1.87.

### Allochthonous humic-like fluorescent dissolved organic matter

A general linear relationship between FDOM_H_ and AOU in intermediate and deep layers, which is indicative of the *in situ* production of FDOM_H_ during the microbial degradation of organic matter, has been observed throughout the open ocean^18–20,36,37^. In this study, the linear relationship between FDOM_H_ and AOU was also evident in the deep layer (>1000 m) along the 160 °E transect (FDOM_H_ = 1.54 × 10^–5^ × AOU + 2.17 × 10^–3^, *n* = 46, adjusted *R*^2^ = 0.93, *p* < 0.01). However, many samples in the mesopelagic layer (200–1000 m) did not follow the linear relationship observed in the deep layer but exhibited deviations from this linear relationship at high levels of FDOM_H_ (Fig. 2a). This deviation from the linear relationship signifies the lack of involvement of the *in situ* process and corresponds to allochthonous FDOM_H_^18^. Thus, in this study, allochthonous FDOM_H_ is defined as FDOM_H_* and is estimated using FDOM_H_, AOU, and the linear regression equation observed in the deep layer as follows:

1 $${{{\rm{FDOM}}}_{{\rm{H}}}}^{\ast }={{\rm{FDOM}}}_{{\rm{H}}}\mbox{--}(1.54\times {10}^{-5}\times {\rm{AOU}}+2.17\times {10}^{-3})$$

### Iron solubility

It has been reported that Fe(III) solubility is linearly related to the FDOM_H_ fluorescence intensity in intermediate and deep waters but not in surface waters^21,23,41,42^. Such differences in these relationships are likely due to the occurrence of organic ligands (e.g., siderophores and saccharides) other than FDOM_H_ in surface waters. Thus, using a previously published dataset^21^, the linear regression between Fe(III) solubility and FDOM_H_ fluorescence intensity in quinine sulfate units (QSU) was determined for the deep waters (>1000 m) of the western subarctic Pacific gyre and the basin of the Sea of Okhotsk collected in 2000 during the R/V *Mirai* cruise (MR00) (Supplementary Fig. 4). Because the instrument-specific response of the spectrofluorometer used in the previous study^21^ was not corrected, the regression equation in Supplementary Fig. 4 could not be directly applied to this study.

Therefore, to determine the calibration factor between the two fluorescence units, namely, the previously reported QSU^21^ and the RU_320_ used in this study, we compared the FDOM_H_ fluorescence in QSU and RU_320_ using samples in the deep layer. For this comparison, two stations located in the western subarctic Pacific gyre and in the basin of the Sea of Okhotsk were selected from each cruise (Supplementary Fig. 5a). Although the observations in this study (MU14) were conducted 14 years after those of the previous study (MR00), the vertical profiles of AOU in the deep layer were almost identical between the two observations (Supplementary Fig. 5b,c). Additionally, the linear relationship between the AOU values in the deep layer of the two cruises is evident, with a slope of almost one (Supplementary Fig. 5d), indicating that the water mass was observed to have the same biogeochemical characteristics in both cruises, thus allowing one to make a calibration factor between RU_320_ and QSU using the relationship between the FDOM_H_ values of the deep layer observed in both cruises (Supplementary Fig. 6).

The conversion factor from FDOM_H_ with units of RU_320_ to Fe(III) solubility with units of nM was achieved using the slope (± a standard deviation) of two relationships, namely, FDOM_H_ [RU_320_] *versus* FDOM_H_ [QSU] (Supplementary Fig. 6) and FDOM_H_ [QSU] *versus* Fe(III) solubility [nM] (Supplementary Fig. 4), as follows:

2 $${\rm{Fe}}({\rm{III}}){\rm{solubility}}[{\rm{nM}}]=200(\pm \,11)\times 0.481(\pm \,0.041)\times {{\rm{FDOM}}}_{{\rm{H}}}[{{\rm{RU}}}_{320}]$$

The estimated value (96.2 ± 9.7) was applied as the conversion factor from FDOM_H_ [RU_320_] to Fe(III) solubility [nM] in this study. The bulk Fe(III) solubility and allochthonous Fe(III) solubility (Fe(III) solubility*) were estimated using the conversion factor with the fluorescence intensity of bulk FDOM_H_ and FDOM_H_*, respectively.

### Ocean data view parameters

Ocean Data View (ODV; http://odv.awi.de/)^59^ was used to produce the basin-scale distributions of each parameter in Figs. 1 and 4 and Supplementary Figs. 1–3. Although high levels of Fe_d_, FDOM_H_, and FDOM_H_* were observed in the shelf region of the Sea of Okhotsk (9.1 nM, 0.020 RU_320_, and 0.018 RU_320_, respectively), the highest ends of the color scales were set to 5 nM for the Fe_d_ concentration (Fig. 1b) and to 0.01 RU_320_ for FDOM_H_ (Fig. 1c) and FDOM_H_* (Fig. 1d) for better visualization. The lowest end of the color scale was set to 0 for FDOM_H_* (Fig. 1d) even though negative values were evident, particularly in surface waters. While high concentrations of colloidal Fe and allochthonous FDOM_H_-Fe complexes were also observed in the shelf region of the Sea of Okhotsk (up to 8.4 nM and 1.8 nM, respectively), as shown in Fig. 4 and Supplementary Fig. 3; the highest end of the color scale was set to 0.7 nM for both species in the figures.

## Supplementary Information

Supplementary Information.

## Data Availability

The datasets presented in the current study are available from the corresponding authors upon reasonable request.
