# Supplementary material for: Shelf humic substances as carriers for basin-scale iron transport in the North Pacific
Source: Sci Rep. 2020 Mar 11;10:4505. doi: 10.1038/s41598-020-61375-7 (PMC7066155; doi:10.1038/s41598-020-61375-7)
Supplement: Supplementary file 1 — Supplementary Information. [file 41598_2020_61375_MOESM1_ESM.pdf]

Scientific Reports

Supplementary Information for

Shelf humic substances as carriers for basin-scale iron transport in the North Pacific

Youhei Yamashita[1]*, Jun Nishioka[2]*, Hajime Obata[3], and Hiroshi Ogawa[3]

[1]Faculty of Environmental and Earth Science, Hokkaido University, Sapporo, Japan
[2]Pan-Okhotsk Research Center, Institute of Low Temperature Science, Hokkaido University, Sapporo, Japan
[3]Atmosphere and Ocean Research Institute, The University of Tokyo, Kashiwa, Japan

*Correspondence and requests for materials should be addressed to Y. Y. (email: yamashiy@ees.hokudai.ac.jp) and J. N. (email: nishioka@lowtem.hokudai.ac.jp)

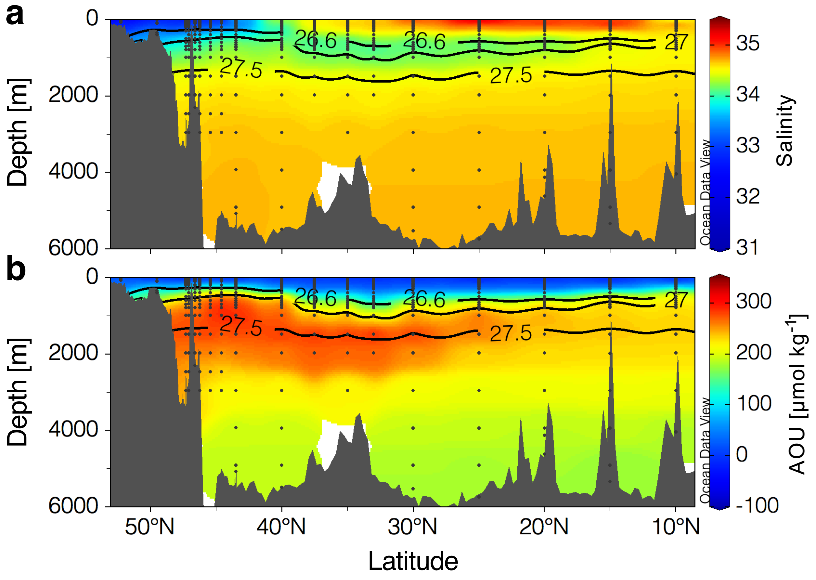

Supplementary Figure 1. Basin-scale distributions of salinity (a) and AOU (b). The solid lines in the figures indicate the contours of $26.6\sigma_\theta$, $27.0\sigma_\theta$, and $27.5\sigma_\theta$, and $26.6–27.0\sigma_\theta$ and $27.0–27.5\sigma_\theta$ correspond to upper and lower intermediate water, respectively.

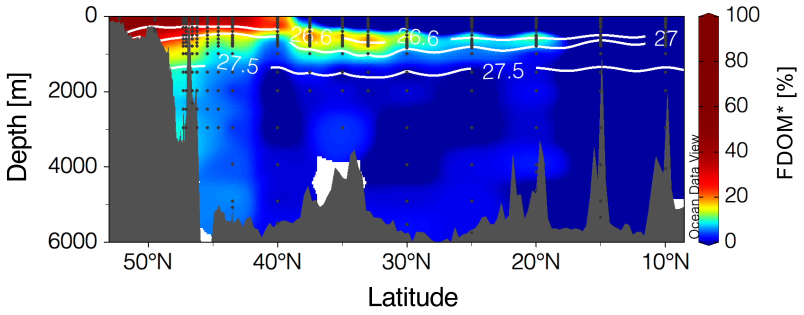

Supplementary Figure 2. Basin-scale distribution of the contribution of allochthonous $FDOM_H$ ($FDOM_H$*) to bulk $FDOM_H$ (%) from the shelf of the Sea of Okhotsk to the subtropical North Pacific. The solid lines in the figure indicate contours of 26.6σ_θ, 27.0σ_θ, and 27.5σ_θ, and 26.6–27.0σ_θ and 27.0–27.5σ_θ correspond to upper and lower intermediate water, respectively.

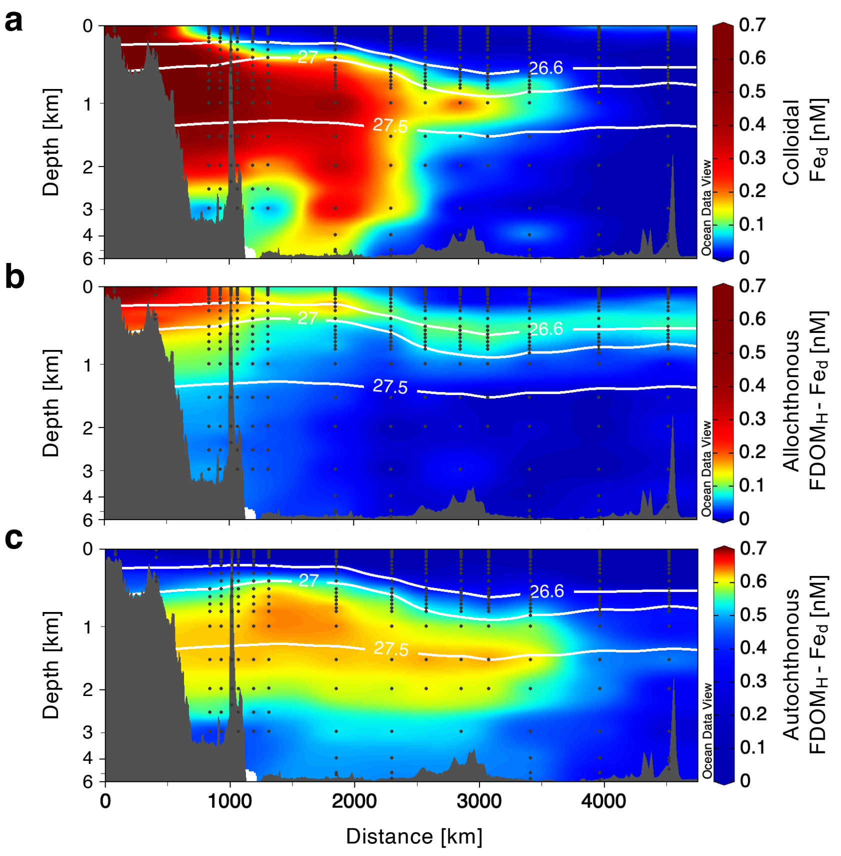

Supplementary Figure 3. Basin-scale distribution of chemical species of Fe_d from the northernmost station on the shelf of the Sea of Okhotsk to the subtropical North Pacific (20°N). a, Colloidal Fe (nM). **b**, Allochthonous $FDOM_H$-Fe complexes (nM). **c**, Autochthonous $FDOM_H$-Fe complexes (nM). The solid white lines in the figures represent the 26.6σ_θ, 27.0σ_θ, and 27.5σ_θ contours, and 26.6–27.0σ_θ and 27.0–27.5σ_θ correspond to upper and lower intermediate water, respectively. The values of 0–2 km are stretched out on the y-axes.

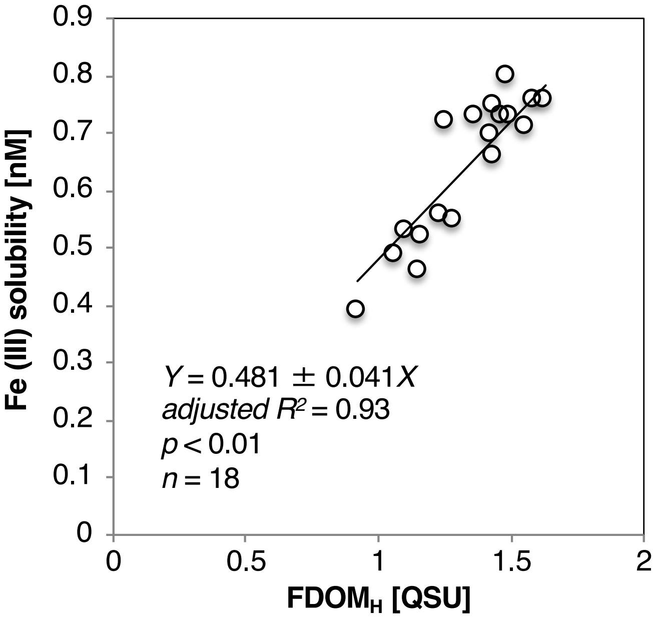

Supplementary Figure 4. Relationship between $FDOM_H$ and Fe(III) solubility in the deep layer of the Sea of Okhotsk and western subarctic Pacific gyre. The data used in the figure were derived from Tani et al. (2003)[21].

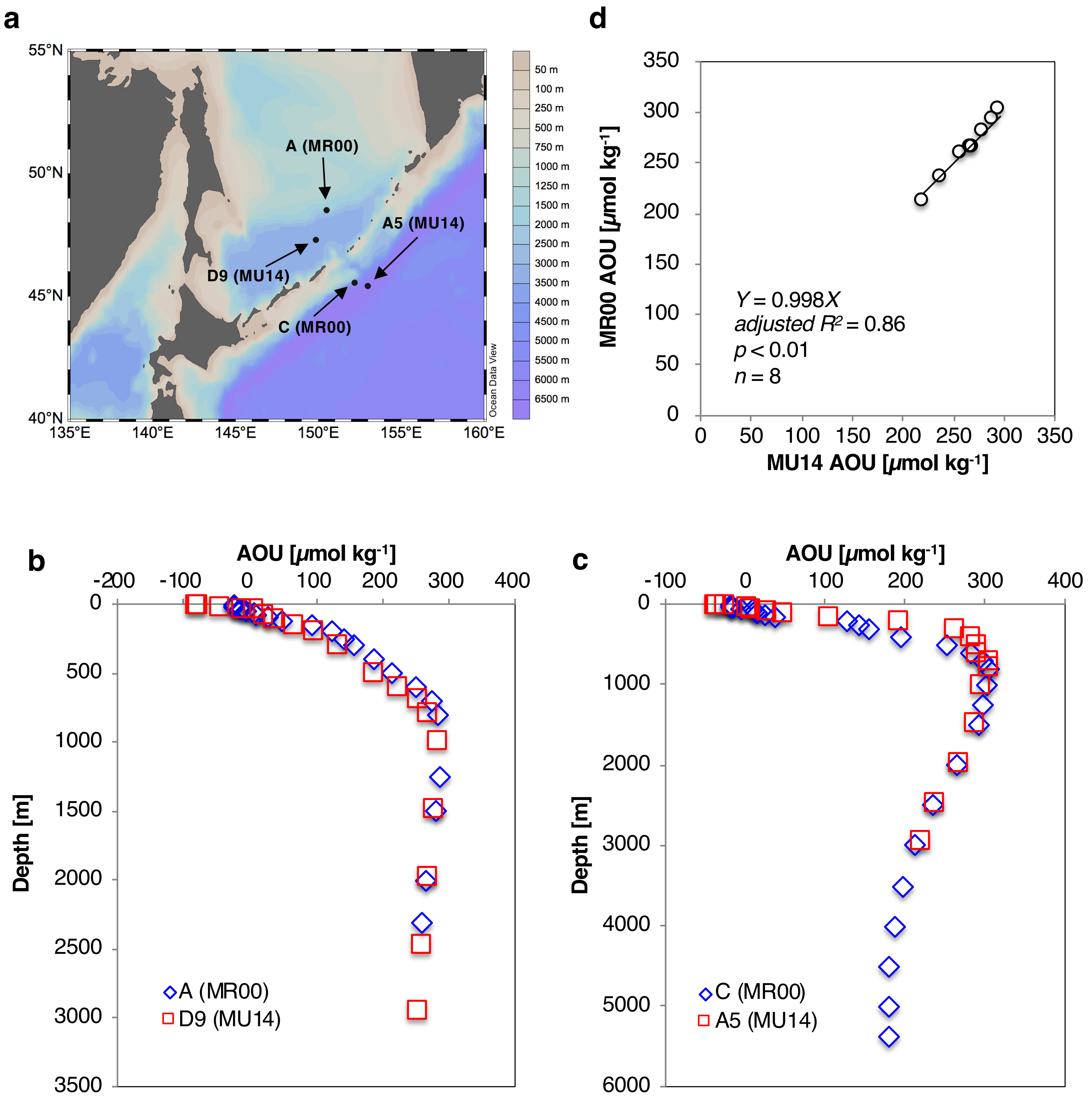

Supplementary Figure 5. Comparison of AOU between two cruises carried out in 2000 (MR00)[21] and 2014 (MU14). **a**, Station locations. **b**, Vertical profiles of AOU in the Sea of Okhotsk. **c**, Vertical profiles of AOU in the western subarctic Pacific gyre. **d**, Relationship of AOU in the deep layer between MU14 and MR00.

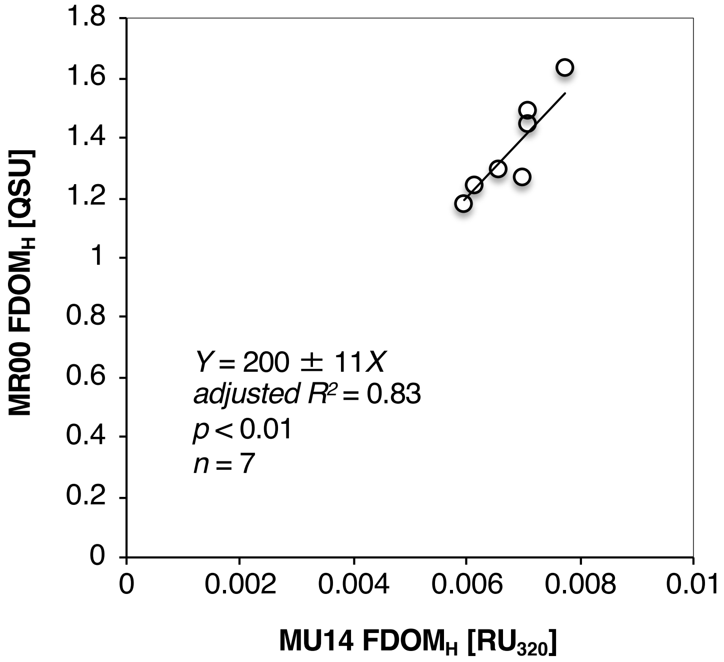

Supplementary Figure 6. Relationship of FDOM$_H$ in the deep layer of the Sea of Okhotsk and the western subarctic Pacific gyre between 2014 (MU14) and 2000 (MR00)[21].
